# Nurse-Led Digital Interventions for Patients with Multiple Sclerosis: A Scoping Review

**DOI:** 10.3390/medsci14020321

**Published:** 2026-06-15

**Authors:** Gianluca Azzellino, Patrizia Vagnarelli, Luca Mengoli, Ernesto Aitella, Mauro Passamonti, Lia Ginaldi, Massimo De Martinis

**Affiliations:** 1 Department of Life, Health and Environmental Sciences, University of L’Aquila, 67100 L’Aquila, Italy; ernesto.aitella@graduate.univaq.it (E.A.); lia.ginaldi@univaq.it (L.G.); 2 Complex Operational Unit, Adriatic District Area, AUSL 04 Teramo, 64100 Teramo, Italy; patrizia.vagnarelli@aslteramo.it (P.V.); mauro.passamonti@aslteramo.it (M.P.); 3 Long-Term Care Unit, “Maria SS. dello Splendore” Hospital, AUSL 04 Teramo, 64021 Giulianova, Italy; luca.mengoli@aslteramo.it; 4 Allergy and Clinical Immunology Unit, Center for the Diagnosis and Treatment of Osteoporosis, AUSL 04 Teramo, 64100 Teramo, Italy; 5 UniCamillus-Saint Camillus International University of Health Sciences, 00131 Rome, Italy

**Keywords:** multiple sclerosis, nurse-led interventions, telenursing, digital health, telehealth, self-management, mobile health (mHealth)

## Abstract

**Background:** Multiple sclerosis (MS) is a condition that requires long-term, multidisciplinary management. The growing digital transformation in healthcare has highlighted the central role of nurses in supporting key aspects such as patient self-management, continuity of (at home) care, and patient empowerment. However, evidence on nurse-led digital interventions in MS remains fragmented. **Objective:** To map the available literature on nurse-led digital interventions in MS, focusing on the role of nurses, clinical outcomes, and research gaps. **Methods:** The review was conducted using the methodological framework of the Joanna Briggs Institute (JBI) and the PRISMA-ScR checklist. A systematic search was performed in PubMed, Scopus, Web of Science, and CINAHL. Studies were included if they described digital or telehealth interventions led or coordinated by nurses in patients with MS. **Results:** A total of 12 studies published between 2015 and 2025 met the inclusion criteria. Four main thematic areas were identified: (1) telenursing and empowerment-based interventions; (2) mobile and web-based patient self-management programs; (3) digital systems for monitoring and integrated care pathways; and (4) digital interventions targeting symptom management and psychosocial outcomes. Across the studies, nurse-led digital interventions were associated with improvements in self-management, treatment adherence, self-efficacy, and health-promoting behaviors. Positive effects were also reported on clinical outcomes such as fatigue, sleep quality, and balance, as well as on psychosocial variables including quality of life, coping strategies, and emotional well-being. Furthermore, the identified systems, in general, contributed to enhanced continuity of care, patient engagement, and organizational efficiency. **Conclusions:** Nurse-led digital interventions represent a promising approach in the management of patients with multiple sclerosis, supporting both clinical and psychosocial outcomes while enhancing continuity of care. However, the current evidence base remains limited by small sample sizes, heterogeneity of interventions, and short follow-up periods. Future research should prioritize multicenter randomized studies with larger samples and long-term follow-up to strengthen the evidence. Additionally, the integration of digital interventions into routine clinical practice, along with targeted training for nurses, is essential to ensure sustainability, accessibility, and equitable implementation. Further studies should also explore cost-effectiveness and the impact on caregivers and long-term quality of life.

## 1. Introduction

Multiple sclerosis is a chronic autoimmune disease affecting the central nervous system and is one of the leading causes of neurological disability among young adults, with its incidence typically peaking around the age of thirty [1,2]. The disease is characterized by a highly heterogeneous clinical presentation, including motor, cognitive, and sensory impairments. Among the various symptoms, fatigue is recognized as one of the most common and disabling manifestations, significantly affecting daily activities, quality of life, and patient autonomy [2,3,4,5].

The management of patients with multiple sclerosis represents a major challenge for healthcare systems due to the chronic and progressive nature of the disease. Effective care requires a multidisciplinary and long-term approach capable of addressing not only clinical needs but also psychosocial and educational aspects throughout the disease trajectory. In addition, the burden of care often extends beyond patients to caregivers, who frequently experience unmet support needs and increased psychological and organizational strain [6,7]. These challenges became even more evident during the COVID-19 pandemic, which exposed limitations in healthcare accessibility and highlighted the importance of reorganizing care models to ensure continuity of care and patient support [8,9].

Within this context, nurses play a central role in the management of chronic conditions such as multiple sclerosis. Nursing interventions include therapeutic education, symptom monitoring, psychosocial support, and the promotion of self-management and patient empowerment, all of which contribute to improving health outcomes and continuity of care [1,5]. The nursing role extends across all stages of the care pathway, encompassing informational support, coordination of multidisciplinary care, and caregiver assistance, and is thus a key component of integrated and patient-centered care models [10].

In recent years, the digital transformation of healthcare systems has introduced innovative models of care delivery, including telemedicine, mobile health applications, educational platforms, telemonitoring, and telenursing. The adoption of these digital tools in multiple sclerosis care has increased considerably, particularly during and after the COVID-19 pandemic [9,11,12].

Digital health technologies offer several potential benefits, including improved access to healthcare services, continuous symptom monitoring, enhanced therapeutic education, and greater patient engagement and empowerment [6,13,14]. In particular, mobile health (mHealth) solutions have emerged as promising tools for supporting disease patient self-management and facilitating communication between patients and healthcare professionals [15].

Current evidence suggests that digital interventions may contribute to reducing fatigue and improving psychological well-being and overall patient experience in individuals with multiple sclerosis [3,16]. Furthermore, technology-supported educational and self-management interventions appear to improve treatment adherence and continuity of care, with nurses frequently assuming a central role in education, coordination, and patient support processes [5,17,18,19]. The integration of telecare and other digital nursing interventions into care pathways may also reduce geographical barriers, increase patient satisfaction, strengthen trust in healthcare systems, and improve continuity of care [6,7,19].

Despite the growing implementation of digital technologies in multiple sclerosis management, the available evidence remains heterogeneous and does not consistently highlight the specific contribution of nurses within digital care pathways. Although recent studies have begun to investigate the use of digital technologies in nursing practice and their potential to improve care organization, information management, and quality of care, further research is still needed to better understand their effectiveness and implementation in clinical settings [20]. Moreover, much of the existing literature primarily focuses on technological outcomes, while the role of nurses in delivering and coordinating digital interventions remains underexplored [5,6,16]. In addition, existing evidence is fragmented across different digital intervention modalities, including telemedicine, mobile applications, web-based platforms, and telemonitoring systems. Furthermore, the specific nursing activities involved in these interventions have not been consistently synthesized, and the heterogeneity of intervention characteristics and outcome measures limits a comprehensive understanding of their implementation and reported outcomes in multiple sclerosis care.

Therefore, a comprehensive synthesis of the available evidence is needed to better clarify the types of digital interventions used in multiple sclerosis care, including mobile applications, telemonitoring systems, and videoconferencing platforms, as well as their clinical, educational, and psychosocial outcomes [13,19]. Understanding these aspects may support the development of evidence-based nursing practices and contribute to the advancement of digitally integrated and patient-centered models of care, which are increasingly shaping the future of nursing practice and community-based healthcare organization [20,21,22]. Given the heterogeneity of intervention modalities, nursing roles, study designs, and outcome measures reported in the literature, a scoping review was considered the most appropriate approach to map the available evidence and provide a comprehensive overview of nurse-led digital interventions in multiple sclerosis care.

Accordingly, this study aims to map and synthesize the available evidence regarding digital nursing interventions for patients with multiple sclerosis, with a particular focus on educational content, clinical outcomes, delivery methods, and the role of nurses in their implementation.

## 2. Materials and Methods

The scoping review was conducted following the methodological framework proposed by the Joanna Briggs Institute (JBI) [23], which is appropriate for mapping the extent, nature, and characteristics of the available evidence on a given topic [24]. The manuscript was prepared in accordance with the Preferred Reporting Items for Systematic Reviews and Meta-Analyses extension for Scoping Reviews (PRISMA-ScR) checklist [25]. Considering the aim of examining the range and nature of the available evidence on digital interventions delivered by nurses for people with multiple sclerosis, a scoping review was deemed the most appropriate methodology [24]. The review protocol was prospectively registered on the Open Science Framework (OSF Registries; https://doi.org/10.17605/OSF.IO/4TK7Q). The review process was developed a priori in accordance with established methodological guidance to ensure rigor and transparency.

The research question guiding the review was:

What evidence is available on digital interventions conducted by nurses for people with multiple sclerosis, including their characteristics and reported outcomes?

To address this question, the PCC (Population, Concept, Context) framework was applied, as recommended by the Joanna Briggs Institute [23].

- Population: Studies including adults (≥18 years) with multiple sclerosis and/or their caregivers were considered eligible. Studies involving pediatric populations were excluded.
- Concept: The review included digital interventions conducted, delivered, designed, or coordinated by nurses, such as telemedicine, eHealth, mHealth, mobile applications, and web-based or digital platforms. Studies were included only when nurses had an active and identifiable role in the design, delivery, coordination, education, monitoring, counseling, or follow-up of the intervention. Interventions were classified as nurse-led when the nursing contribution represented an integral component of the digital intervention and could be clearly identified from the study description. Interventions not involving digital components or without nursing involvement were excluded.
- Context: Studies conducted in any healthcare setting were included, including hospital, community, home-based, and remote care environments.

### 2.1. Search Strategy and Databases

A comprehensive search strategy combining controlled vocabulary (e.g., MeSH terms and CINAHL Headings) and free-text keywords was developed. The main concepts included “multiple sclerosis”, “nursing”, and “digital health”, along with related terms such as “telemedicine”, “telehealth”, “eHealth”, “mHealth”, “mobile applications”, and “remote monitoring”. These terms were combined using Boolean operators (AND, OR). The search was conducted in the following electronic databases: PubMed, Scopus, Web of Science, and CINAHL. The final search was performed on 15 March 2025. No time limits were applied, and only studies published in English were considered. The full search strategies for each database are reported in Supplementary File S1.

### 2.2. Study Selection and Screening Process

**Figure 1:**
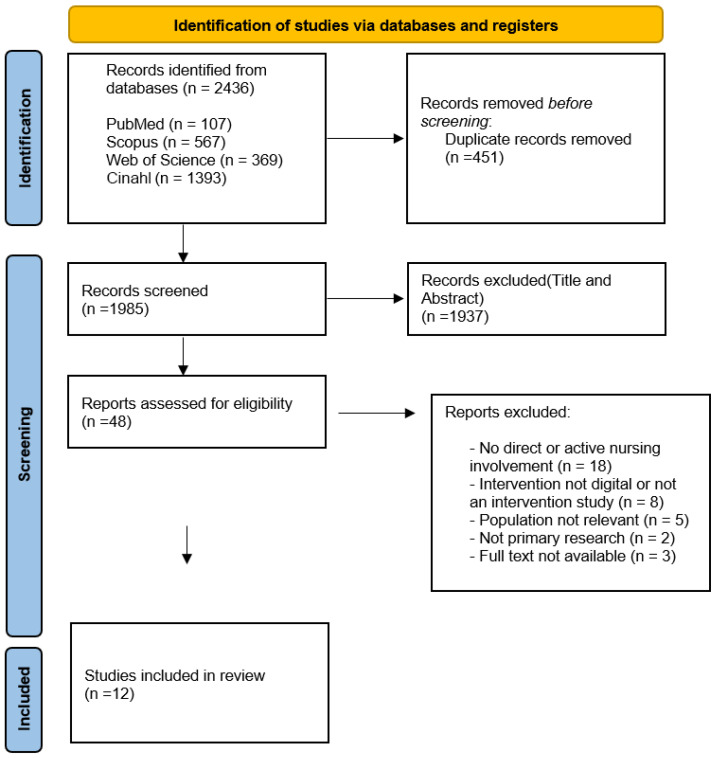
1-ScR flow diagram of the study selection process. Literature searches were conducted in PubMed, Scopus, Web of Science, and CINAHL.

The study selection process was conducted in two phases. First, two reviewers (GA and MD) independently screened the titles and abstracts of all retrieved records based on predefined inclusion and exclusion criteria. Subsequently, full-text articles were assessed to confirm eligibility. The screening process was performed manually by two independent reviewers, with records managed using *Zotero reference management software* (version 7.0.21, Corporation for Digital Scholarship, Vienna, VA, USA) for organization and duplicate removal. Any discrepancies were resolved through discussion or, when necessary, with the involvement of additional reviewers (PV, LG, MP). The study selection process is reported in the PRISMA-ScR flow diagram (Figure 1).

### 2.3. Inclusion and Exclusion Criteria

Eligibility criteria were defined a priori. Studies were included if they were primary research (quantitative, qualitative, or mixed-methods) investigating digital interventions conducted, delivered, designed, or coordinated by nurses and targeting adults (≥18 years) with multiple sclerosis and/or their caregivers, in any healthcare setting (hospital, community, home-based, or remote). Only studies published in English were considered, with no time restrictions applied. Studies were excluded if they did not involve nurses directly, if the intervention did not include a digital component (i.e., exclusively face-to-face), if they focused on pediatric populations (<18 years), or if they were reviews, editorials, commentaries, conference abstracts, or study protocols.

### 2.4. Data Extraction

Data were extracted from the included studies using a structured data charting form developed in Microsoft Excel prior to the review. The form was designed to ensure a systematic and consistent collection of relevant information across studies. Extracted data included author and year of publication, country, main theme, study design, sample characteristics, type of digital intervention, nursing role, mode of delivery, intervention description, key findings, and measured outcomes. Data extraction was performed independently by two reviewers (GA and PV). To ensure accuracy and consistency, a third reviewer (MD) independently verified the extracted data. Any discrepancies were resolved through discussion and consensus.

### 2.5. Data Synthesis

The results were narratively synthesized into the following main thematic categories, based on the characteristics and outcomes of the included studies:

- Telenursing and empowerment-based interventions;
- Mobile and web-based self-management interventions;
- Digital systems for monitoring and integrated care;
- Digital interventions for symptom management and psychosocial outcomes.

In accordance with the Joanna Briggs Institute methodology for scoping reviews, a quantitative synthesis of the results was not performed [23]. Additionally, a critical appraisal of the methodological quality of the included studies was not undertaken, consistent with the nature and purpose of scoping reviews [23,24].

## 3. Results

The database search identified a total of 2436 records. After removing duplicates (*n* = 451), 1985 titles and abstracts were screened. Of these, 48 studies were considered potentially eligible and were retrieved in full text. Subsequently, all 48 articles were assessed for final inclusion. At the end of the selection process, 12 studies were included in the review. The identification, screening, and selection process is illustrated in the PRISMA flow diagram (Figure 1).

### 3.1. Characteristics of the Included Studies

**Figure 2:**
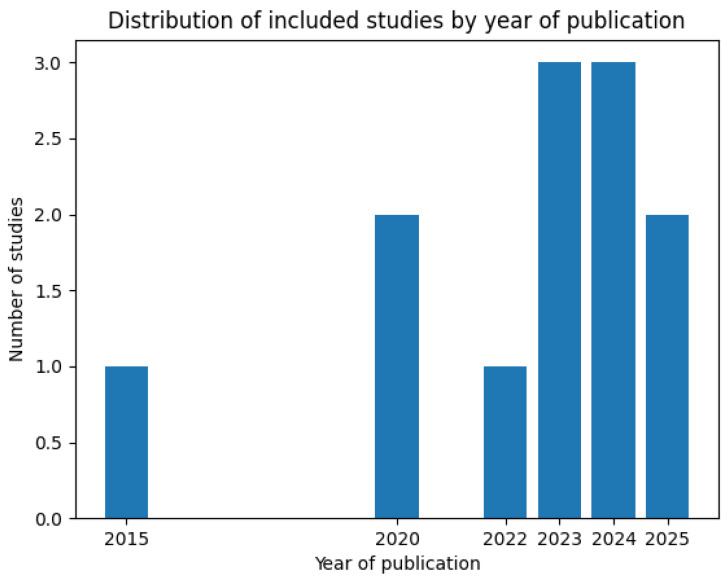
Distribution of included studies by year of publication.

A total of 12 studies were included in this review, published between 2015 and 2025 (Figure 2). Overall, a progressive increase in publications was observed over time, with a higher concentration of studies in recent years, reflecting the growing interest in digital health interventions for people with multiple sclerosis (MS). The main characteristics of the included studies are summarized in Table 1.

Most studies were conducted in Iran (*n* = 6; 50.0%), followed by Turkey (*n* = 2; 16.7%), with single contributions from China, the Netherlands, Egypt, and the United States, highlighting a geographically diverse but regionally concentrated body of evidence.

Regarding study design, randomized controlled trials (RCTs) [1,26,27,32,35] accounted for (*n* = 5; 41.7%) of the included studies, while quasi-experimental designs [19,29,30,31,34] accounted for (*n* = 5; 41.7%) and were equally represented. The remaining studies included pilot, observational, and implementation studies [28,33] (*n* = 2; 16.7%) [28,33].

Sample sizes varied considerably, ranging from 20 to 581 participants, indicating substantial heterogeneity in study scale. All included studies examined digital or technology-based interventions in patients with multiple sclerosis; however, considerable heterogeneity emerged in the type and complexity of interventions. These ranged from mobile applications and web platforms to structured telenursing programs, often integrating different digital tools such as smartphone apps, messaging systems (e.g., WhatsApp, WeChat), videoconferencing platforms (e.g., Zoom), and telephone follow-up [27,29,30,34].

In most cases, interventions were delivered through a mixed approach (*n* = 10; 83.3%), combining asynchronous components, such as educational content, mobile applications, or digital platforms, with synchronous interactions, including phone calls, video consultations, online sessions, or in-person meetings [1,26,27,29,30,31,32,33,34,35].

**Figure 3:**
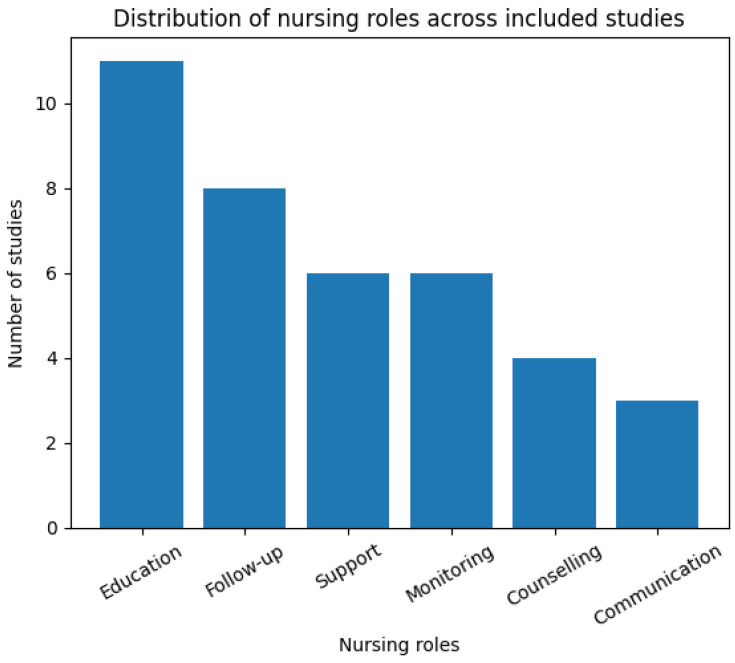
Distribution of nursing roles across the included studies.

The role of nurses was central across the included studies (Figure 3). Nurses were involved in a range of activities, including education, counseling, monitoring, and follow-up, and in some cases also contributed to the development or implementation of intervention content. Education was the most frequently reported nursing activity, followed by follow-up and monitoring, while counselling and communication were less commonly described. Less frequently reported roles included guidance, content development, coordination, implementation, and feedback. In telenursing-based interventions, nursing staff were involved in delivering educational interventions, providing counselling, and conducting follow-up, using digital tools such as phone calls, WhatsApp, and online platforms.

In terms of outcomes, the included studies assessed a wide range of variables, including fatigue, quality of life, self-management, treatment adherence, self-efficacy, balance, sleep quality, coping strategies, and health-promoting behaviors.

### 3.2. Main Results

The findings of the included studies were narratively synthesized into four main thematic categories.

#### 3.2.1. Telenursing and Empowerment-Based Interventions

Several studies highlighted the effectiveness of telenursing interventions in promoting patient empowerment, self-management, and health-related behaviors among individuals with multiple sclerosis. Nurse-led programs delivered through various digital modalities showed significant improvements in self-management and disease-related knowledge [1,33]. Telenursing educational interventions also demonstrated positive effects on health-promoting behaviors, including nutrition, stress management, and lifestyle modification [27]. Moreover, structured online support programs contributed to improvements in fatigue, sleep quality, and physical activity levels, although effects on quality of life were sometimes partial [34]. Similarly, mobile-assisted nursing interventions in specific populations, such as postpartum women, improved self-efficacy and self-management behaviors [30]. Overall, these findings suggest that telenursing interventions play a central role in enhancing patient engagement and supporting continuity of care in individuals with multiple sclerosis.

#### 3.2.2. Mobile and Web-Based Self-Management Interventions

A substantial number of studies focused on mobile and web-based interventions designed to support self-management and adherence. Smartphone applications based on structured care models significantly improved treatment adherence and self-efficacy among patients with multiple sclerosis [19]. Similarly, mobile-based self-care programs demonstrated positive effects on clinical outcomes such as balance and daily functioning [32]. Educational mobile applications, such as MobilMS, were associated with improvements in quality of life, although no significant effects on symptom severity or treatment adherence were observed [35]. In addition, both smartphone-based and face-to-face educational interventions were found to be effective in improving quality of life and coping skills, with no significant differences between delivery modalities [26]. Web-based self-management platforms also showed improvements in patient compliance and knowledge, supporting the role of digital tools in enhancing long-term disease management [33].

#### 3.2.3. Digital Systems for Monitoring and Integrated Care Pathways

Several studies described digital systems designed to support monitoring, communication, and coordination of care for people with multiple sclerosis. Web-based platforms such as MSmonitor enabled symptom tracking, standardized assessments, and direct communication with healthcare professionals, contributing to improved quality of life and supporting multidisciplinary care [28]. Similarly, web-based interventions supported by communication tools facilitated patient education, symptom monitoring, and interaction with healthcare providers, leading to improved self-management compliance and increased patient knowledge [33]. Other mobile-based interventions also included monitoring and communication features, such as medication tracking and follow-up, although these were primarily focused on self-management rather than fully integrated care pathways [19,35]. Overall, digital systems appear to enhance monitoring, communication, and continuity of care in multiple sclerosis management, particularly when supported by nursing and multidisciplinary collaboration.

#### 3.2.4. Digital Interventions for Symptom Management and Psychosocial Outcomes

Digital interventions demonstrated consistent effects on both symptom management and psychosocial outcomes among individuals with multiple sclerosis. Several studies reported improvements in key clinical symptoms, particularly fatigue and sleep quality. Mobile health self-care training programs significantly reduced fatigue levels [31], while nurse-led online support interventions improved fatigue, sleep quality, and physical activity levels, although quality-of-life improvements were only partial [34]. Similarly, mindfulness-based and sleep education programs delivered via videoconference contributed to improvements in sleep quality, mindfulness, and overall well-being [29]. In addition to symptom-related outcomes, multiple studies highlighted positive effects on psychosocial variables. Improvements in quality of life were consistently reported across different types of digital interventions, including mobile applications and web-based systems [26,28,35]. Furthermore, interventions based on mobile applications and continuous care models significantly enhanced self-efficacy and treatment adherence [19], while mobile-assisted nursing programs improved self-efficacy and self-management behaviors in specific populations, such as postpartum women [30]. Other psychosocial outcomes, including coping skills, mindfulness, and emotional well-being, were also positively influenced by digital interventions. For instance, both smartphone-based and face-to-face educational interventions improved coping strategies [26], while videoconference-based programs enhanced mindfulness and psychological well-being [29]. Overall, these findings suggest that digital nursing interventions can positively impact both clinical and psychosocial domains, particularly fatigue, sleep, self-efficacy, quality of life, and coping strategies, although some variability in effectiveness was observed across studies and outcomes.

## 4. Discussion

The results of this scoping review highlight how nurse-led digital interventions in multiple sclerosis are not limited to facilitating access to healthcare services, but also play a central role in promoting disease self-management, treatment adherence, and psychosocial support. In line with the findings, the nursing role appears to be broad and multifaceted, encompassing education, monitoring, counseling, and continuous follow-up activities. From a clinical perspective, several studies included in the review reported improvements in self-management behaviors, self-efficacy, and treatment adherence. These findings suggest that telenursing programs and mobile health-based interventions may offer potential benefits for patients with multiple sclerosis. These findings are consistent with the international literature, which recognizes nursing interventions as playing a key role in enhancing self-efficacy and well-being among patients with chronic conditions [36,37]. Similarly, recent systematic reviews have highlighted the effectiveness of telehealth interventions in supporting self-management in chronic diseases, including multiple sclerosis [6].

From a broader perspective, digital health interventions have been increasingly explored as promising approaches for the management of chronic diseases, particularly because of their potential to enhance patient engagement, personalize care, and improve the efficiency of healthcare systems [38]. The effectiveness of these interventions may be explained by their multidimensional nature. Nurse-led digital interventions combine educational, behavioral, and relational components, promoting active patient engagement and greater continuity of care. This approach is particularly relevant in multiple sclerosis, a condition characterized by a variable course and complex long-term care needs.

Another important finding concerns the role of digital systems in supporting monitoring and integrated care pathways. Web-based platforms and telemonitoring systems, such as MSmonitor and other digital tools, have been shown to improve communication between patients and healthcare professionals, symptom monitoring, and continuity of care. These findings are consistent with previous studies highlighting how digital technologies can enhance safety, traceability, and coordination of care [16,39,40]. Furthermore, the use of telemedicine has been associated with reduced healthcare costs and improved access to services for patients with multiple sclerosis [41]. In this context, the value of integrated interventions and continuity of care during the post-discharge period also emerges strongly. Recent evidence suggests that models based on combined interventions and early activation of home care, with the active involvement of coordinating nurses, can significantly reduce hospital readmissions and improve clinical outcomes [18]. These findings further reinforce the role of nurse-led interventions in managing care transitions and strengthening the hospital–community connection.

Although the included studies reported generally positive outcomes, several challenges emerged, including intervention heterogeneity, limited telemonitoring implementation, difficulties in remote clinical assessment, and limited generalizability due to small monocentric samples. Interestingly, several studies identified during screening were excluded because nurses were not involved in the intervention, suggesting that the role of nurses within digital multiple sclerosis care remains relatively underrepresented in the current literature and warrants further investigation. In addition, previous literature highlighted barriers related to interoperability, privacy, data security, and professional regulations in digital health implementation [42]. Furthermore, the risk of digital inequalities remains significant, as access to technologies and digital literacy are not equally distributed among patients, a challenge that became particularly evident during the COVID-19 pandemic [43,44].

The COVID-19 pandemic accelerated the adoption of telemedicine and hybrid care models [45,46]. However, this rapid transition also highlighted the need for adequate technological infrastructure and targeted training for healthcare professionals, particularly nurses involved in digital care delivery.

Several included studies reported improvements in quality of life, coping strategies, and emotional well-being. In particular, smartphone-based educational interventions and online support programs demonstrated positive effects on fatigue, sleep quality, self-efficacy, coping skills, and overall quality of life. These findings are consistent with previous literature highlighting the effectiveness of nurse-led digital interventions and online cognitive behavioral approaches in reducing emotional distress and supporting relapse management in patients with multiple sclerosis [47,48,49]. Furthermore, patient co-designed digital applications have shown promising results in promoting self-management and psychological well-being.

Overall, the findings of this review support the value of nurse-led digital interventions in multiple sclerosis management, highlighting their potential to improve both clinical and psychosocial outcomes. However, intervention heterogeneity, methodological limitations, and the lack of shared standards underline the need for further research to strengthen the evidence base and support their integration into clinical practice.

### 4.1. Implications for Clinical Practice

The findings of this review suggest that nurse-led digital interventions may represent a valuable strategy to improve the management of people with multiple sclerosis in clinical practice. In particular, digital interventions may support patient education, enhance treatment adherence, promote self-management, and improve psychosocial well-being through continuous and personalized follow-up. These findings highlight the potential role of nurses in integrating digital health tools into chronic disease management pathways, contributing to more accessible, patient-centered, and continuity-based models of care. However, successful implementation in routine clinical practice requires adequate professional training, digital competencies, and organizational support within healthcare systems.

### 4.2. Limitations

The included studies were characterized by relatively small sample sizes, short follow-up periods, and predominantly monocentric designs, which may limit the generalizability and external validity of the findings. Additionally, the heterogeneity of interventions and outcome measures further complicates the comparability of results across studies. Furthermore, a substantial proportion of the included studies were conducted in Iran, which may limit the transferability of the findings to healthcare systems characterized by different nursing roles, scopes of practice, levels of professional autonomy, and degrees of integration of digital health services. Therefore, the applicability of these findings to other healthcare contexts and nursing systems should be interpreted with caution.

## 5. Conclusions

This scoping review mapped the available evidence on nurse-led digital interventions for people with multiple sclerosis, highlighting their potential to improve self-management, treatment adherence, patient empowerment, and psychosocial well-being. Across the included studies, digital interventions delivered or coordinated by nurses were associated with positive outcomes in several domains, including fatigue management, sleep quality, coping strategies, self-efficacy, and quality of life. Furthermore, digital tools contributed to strengthening continuity of care, patient engagement, and communication between patients and healthcare professionals. Despite these promising findings, the current body of evidence remains limited by methodological heterogeneity, small sample sizes, and short follow-up periods, which restrict the generalizability of results. Additional high-quality multicenter studies are needed to better define the effectiveness, sustainability, and long-term impact of nurse-led digital interventions in multiple sclerosis care. Future research should also explore the integration of digital nursing interventions into routine clinical practice, the development of standardized intervention models, and the role of nurses in increasingly technology-driven healthcare systems. Particular attention should be given to digital accessibility, professional training, and the reduction of health inequalities to ensure equitable and patient-centered care for individuals living with multiple sclerosis.

## Figures and Tables

**Table 1:** Characteristics of included studies (*n* = 12).

Author/Year	Main Theme	Country	Design	Sample (Patients)	Digital Intervention	Nursing Role	Delivery Mode	Intervention Description	Key Findings	Outcomes Measured
Baharian et al. [26]	Comparison of smartphone-based and face-to-face self-care education on quality of life and coping skills in patients with multiple sclerosis	Iran	Randomized controlled trial	60	Smartphone-based self-care education delivered through a mobile application installed on patients’ phones	Education; Guidance; Follow-up; Support	Mixed (smartphone-based mobile application, face-to-face education sessions, and telephone follow-up)	A 4-week self-care educational program delivered either through face-to-face sessions or a smartphone application, including disease education, symptom management, medication guidance, and lifestyle strategies, followed by a 4-week self-care period with weekly telephone follow-up	Both smartphone-based and face-to-face education significantly improved quality of life and coping skills, with no significant difference between the two methods after the intervention	Quality of life (Multiple Sclerosis Impact Scale-29) and coping skills (Multiple Sclerosis Coping Questionnaire)
Bayat et al. [1]	Patient-centered empowerment program through telenursing to improve self-management in people with multiple sclerosis	Iran	Double-blinded randomized clinical trial	90	Telenursing-based empowerment program using telephone, Skype and WhatsApp	Education; Counseling; Follow-up; Support	Mixed (Synchronous Telenursing via phone/Skype and asynchronous support via WhatsApp	A patient centered empowerment program delivered through 5 telenursing sessions over 4 weeks, covering physical, psychological, and social aspects, with individualized counseling based on patient needs and supported by digital communication tools (Skype and WhatsApp)	The telenursing empowerment program significantly improved self-management and related subscales (knowledge and health maintenance behaviors) in patients with multiple sclerosis compared to the control group	Self-management (Multiple Sclerosis Self-Management Scale and its subscales: communication, adherence, support, knowledge, health behaviors)
Dehghani et al. [27]	Effect of telenursing self-care education on health-promoting behaviors in MS patients	Iran	Clinical trial study	68	Telenursing self-care education via: WhatsApp -phone calls -multimedia content	Education; Counseling; Follow-up	Mixed: asynchronous (WhatsApp content) synchronous (phone calls)	Self-care education program delivered over 6 weeks, including topics such as nutrition, exercise, stress management, interpersonal relationships, and self-actualization, using WhatsApp-based materials and regular telephone follow-up sessions	Telenursing education significantly improved health-promoting behaviors in MS patients compared to the control group	Health-promoting behaviors using Walker’s Health-Promoting Lifestyle Profile (HPLP)
Jongen et al. [28]	Development and evaluation of a web-based self-management program (MSmonitor) for multidisciplinary care in multiple sclerosis	Netherlands	Pilot study (observational, retrospective analysis)	581 (subsample analyzed: 105 patients)	An interactive web-based program (MSmonitor) including patient-reported questionnaires, symptom and activity diaries, an e-consult function for communication with healthcare professionals, and a personal e-logbook for self-monitoring and management	Monitoring; Communication; Support; Implementation	Web-based	MSmonitor is an interactive web-based self-management and multidisciplinary care program that allows patients to complete validated questionnaires, track symptoms via diaries, communicate with healthcare professionals through e-consult, and monitor their condition over time	-Use of MSmonitor associated with improved quality of life; -diary usage correlated with fatigue improvement; -self-monitoring supports self-management	Fatigue (measured using the Modified Fatigue Impact Scale-5), quality of life (assessed with the Leeds Multiple Sclerosis Quality of Life scale and the Multiple Sclerosis Quality of Life-54), anxiety and depression (measured using the Hospital Anxiety and Depression Scale), and disability (assessed using the Multiple Sclerosis Impact Profile)
Kazemi et al. [19]	Effect of a smartphone-based continuous care model on treatment adherence and self-efficacy in MS patients	Iran	Quasi-experimental (pre/post-test design)	72	Smartphone application (“MS App”) based on Continuous Care Model	Education; Monitoring; Follow-up; Communication	Mobile health	Continuous Care Model delivered Via A Smartphone application over 2–4 months, including education, monitoring, interaction, and follow-up through multimedia content, chat features, and communication with healthcare providers	Significant Improvement in: -treatment adherence -self-efficacy in the intervention group compared to control	Treatment adherence Self-efficacy
Lorenz et al. [29]	Mindfulness plus sleep education intervention to improve sleep in MS patients	USA	Quasi-experimental pilot study	34	Videoconference -based mindfulness Sleep education program (SleepWell!)	Education; Monitoring; Guidance	Mixed: synchronous videoconference (Zoom sessions) group-based sessions	SleepWell! is an 8-Week multicomponent program combining mindfulness-based stress reduction and sleep hygiene education, delivered via videoconference or onsite group sessions, including guided meditation, behavioral sleep strategies, and group interaction	-Improved sleep efficiency and total sleep time; -improvements in sleep quality, mindfulness, and quality of life; -videoconference delivery feasible and effective	Outcomes included sleep (measured using the Pittsburgh Sleep Quality Index and actigraphy), fatigue (Fatigue Severity Scale), depression (Center for Epidemiologic Studies Depression Scale-Revised), quality of life (Multiple Sclerosis Quality of Life-54), and mindfulness (Cognitive and Affective Mindfulness Scale-Revised)
Rashed et al. [30]	Effect of smartphone application-assisted nursing intervention on breastfeeding self-efficacy in postpartum women with MS	Egypt	Quasi-experimental (case–control)	50	Smartphone application-assisted nursing intervention (Zoom and WhatsApp support)	Education; Counseling; Follow-up	Mixed: synchronous (Zoom sessions) asynchronous (WhatsApp Messages/support)	Mobile application-assisted nursing program including three weekly educational sessions (20–30 min each) delivered via Zoom, supported by WhatsApp follow-up messages, covering breastfeeding, MS management, and postpartum care	The intervention significantly improved breastfeeding self-efficacy and postpartum self-management in women with multiple sclerosis	Breastfeeding self-efficacy (BSES scale) Postpartum MS relapse variables
Roshanghiyas et al. [31]	Mobile health self-care training to reduce fatigue in MS patients	Iran	Quasi-experimental	80	Web-based mobile health education platform	Education; Support; Follow-up	Mixed (asynchronous web-based + synchronous phone follow-up)	Mobile health self-care training delivered via a web-based platform, including weekly educational videos and podcasts, with nurse follow-up through phone calls	Mobile health self-care training significantly reduced fatigue levels in patients with multiple sclerosis compared to the control group	Fatigue (Fatigue Severity Scale—FSS)
Safian et al. [32]	Effect of a mobile-based self-care program on balance in people with multiple sclerosis	Iran	Randomized Controlled Trial (RCT)	72	Mobile App	Education, monitoring, support	Mixed (mobile app, phone follow-up, in-person session)	Mobile-based self-care program delivered via a smartphone application including education on disease management, exercise, and daily living, with ongoing monitoring and telephone follow-up over two months	Mobile-based self-care intervention significantly improved balance in patients with multiple sclerosis compared to the control group	Balance (Tinetti Performance Oriented Mobility Assessment)
Xu et al. [33]	Improving self-management in discharged patients with multiple sclerosis using a web-based intervention	China	Implementation study (quality improvement project)	20	Web-based self-management platform (WeChat-based)	Education; Coordination; Support; Monitoring; Communication	Mixed (web-based Platform, Education, communication via WeChat)	Web-based self-management intervention delivered via a WeChat platform including education, symptom monitoring, medication adherence support, exercise guidance, psychological support, and communication with healthcare providers	The web based self-management intervention significantly improved compliance with self-management practices and enhanced patients’ knowledge and staff awareness	Compliance with self-management best practice criteria
Yalçın et al. [34]	Effect of a nurse-led online support program on fatigue, sleep, and quality of life in MS patients	Turkey	Quasi-experimental study	30	Nurse-led online support program delivered via Zoom (online sessions and follow-up)	Education; Monitoring; Follow-up; Feedback	Mixed: synchronous (live Zoom sessions) synchronous follow-up (phone calls)	A 5-week nurse-led online support program including 10 live educational sessions via Zoom, focusing on fatigue management, lifestyle, and self-management, with weekly follow-up calls to reinforce adherence	The intervention significantly improved: -fatigue -sleep quality -physical activity (step count) while quality of life showed partial improvement	Fatigue (Fatigue Severity Scale) Sleep (Pittsburgh Sleep Quality Index) Quality of life (EQ-5D) Physical activity (step count)
Üstündağ et al. [35]	Effect of a mobile educational application (MobilMS) on symptom management and quality of life in MS patients	Turkey	Randomized Controlled Trial (RCT)	63	Mobile application: MobilMS	Education; Counseling; Follow-up; Content development	Mixed (mobile app-based intervention with remote follow-up via phone/video calls)	A 12-week mobile health educational intervention based on the Information–Motivation–Behavioral Skills model, delivered via the MobilMS app, including disease education, symptom management, medication tracking, and communication with healthcare professionals	The intervention significantly improved quality of life in patients with multiple sclerosis, although no statistically significant effects were observed on symptom severity or treatment adherence; the app was highly accepted and well evaluated by patients	Symptoms (MS-RS checklist), quality of life (MSQoL-54), treatment adherence (MS-TAQ)

## Data Availability

The original contributions presented in this study are included in the article/Supplementary Materials. Further inquiries can be directed to the corresponding authors.

## Supplementary Materials

The following supporting information can be downloaded at: https://www.mdpi.com/article/10.3390/medsci14020321/s1, File S1: Detailed search strategies.
