# Supplementary material for: Nurse-Led Digital Interventions for Patients with Multiple Sclerosis: A Scoping Review
Source: Med Sci (Basel). 2026 Jun 15;14(2):321. doi: 10.3390/medsci14020321 (PMC13302828; doi:10.3390/medsci14020321)
Supplement: Supplementary file 1 [file medsci-14-00321-s001.zip › medsci-4357272-supplementary.pdf]

Supplementary File S1. Detailed search strategies

The search strategies were developed for each database using a combination of controlled vocabulary and free-text terms. The final search was conducted on 15 March 2025.

Pubmed

("Multiple Sclerosis"[Mesh] OR "multiple sclerosis")

AND

("Nursing"[Mesh] OR nurse* OR nursing OR "nursing care" OR "nursing intervention*")

AND

("Telemedicine"[Mesh] OR "Digital Health" OR telemedicine OR telehealth OR eHealth OR mHealth OR "mobile health" OR "mobile application*" OR app* OR "remote monitoring" OR "digital intervention*" OR "online intervention*" OR "web-based")

Scopus

TITLE-ABS-KEY("multiple sclerosis")

AND

TITLE-ABS-KEY(nurs* OR "nursing care" OR "nursing intervention*")

AND

TITLE-ABS-KEY("digital health" OR telemedicine OR telehealth OR ehealth OR mhealth OR "mobile health" OR "mobile application*" OR app* OR "remote monitoring" OR "digital intervention*" OR "online intervention*" OR "web-based")

Web of science

TS=("multiple sclerosis")

AND

TS=(nurs* OR "nursing care" OR "nursing intervention*")

AND

TS=("digital health" OR telemedicine OR telehealth OR ehealth OR mhealth OR "mobile health" OR "mobile application*" OR app* OR "remote monitoring" OR "digital intervention*" OR "online intervention*" OR "web-based")

CINAHL

((MH "Multiple Sclerosis") OR "multiple sclerosis")

AND

((MH "Nursing") OR nurs* OR "nursing care" OR "nursing intervention*")

AND

((MH "Telemedicine") OR telemedicine OR telehealth OR ehealth OR mhealth OR "mobile health" OR "remote monitoring" OR "digital health")
